# Assessment and Management of Pain in Preterm Infants: A Practice Update

**DOI:** 10.3390/children9020244

**Published:** 2022-02-11

**Authors:** Marsha Campbell-Yeo, Mats Eriksson, Britney Benoit

**Affiliations:** 1 School of Nursing, Faculty of Health, Dalhousie University, Halifax, NS B3H 4R2, Canada; 2 Department of Pediatrics, Psychology and Neuroscience, Dalhousie University, Halifax, NS B3H 4R2, Canada; 3 IWK Health, Halifax, NS B3K 6R8, Canada; 4 School of Health Sciences, Faculty of Medicine and Health, Örebro University, SE-701 82 Örebro, Sweden; mats.h.eriksson@oru.se; 5 Rankin School of Nursing, St. Francis Xavier University, Antigonish, NS B2G 2N5, Canada; bbenoit@stfx.ca

**Keywords:** preterm, infant, neonate, NICU, pain, procedural, assessment, management

## Abstract

Infants born preterm are at a high risk for repeated pain exposure in early life. Despite valid tools to assess pain in non-verbal infants and effective interventions to reduce pain associated with medical procedures required as part of their care, many infants receive little to no pain-relieving interventions. Moreover, parents remain significantly underutilized in provision of pain-relieving interventions, despite the known benefit of their involvement. This narrative review provides an overview of the consequences of early exposure to untreated pain in preterm infants, recommendations for a standardized approach to pain assessment in preterm infants, effectiveness of non-pharmacologic and pharmacologic pain-relieving interventions, and suggestions for greater active engagement of parents in the pain care for their preterm infant.

## 1. Introduction

Ubiquitous exposure to acute pain is inevitable for all infants, for most within the initial hour and first day(s) after birth as part of recommended medical care [1]. Infants will undergo a routine intramuscular injection of vitamin K to prevent bleeding [2,3], a heel lance to collect blood for metabolic testing [4], and many will have routine total serum bilirubin screening [5], blood glucose, or other blood tests within the first days of age. Additionally, many infants will receive a preventative vaccine by intramuscular injection (i.e., hepatitis B) shortly after birth, and most children receive recommended immunizations; upwards of 20 intramuscular injections between two to 18 months of age [6].

For infants delivered preterm, the majority requiring neonatal intensive care, pain exposure is even higher. Data from Canadian [7] and European [8,9,10,11] studies demonstrate that infants can undergo anywhere from one to 14 procedures per day when hospitalized in the neonatal intensive care unit (NICU). A review including 18 studies examining pain exposure and analgesic practices across numerous countries conducted by Cruz and colleagues (2016) found that hospitalized neonates were undergoing 7–17 painful procedures per day, with the most common procedures being heel lancing, naso- and endo-tracheal suctioning, venipuncture, and insertion of peripheral venous catheters. Additional routinely performed painful procedures include intubation, chest tube placement, lumbar puncture, insertion of arterial and venous umbilical catheters and peripheral arterial catheters, intramuscular and subcutaneous injections, tape removal, and retinopathy of prematurity eye examinations. Studies in this review reported that infants went without any form of analgesia during painful procedures ranging from 42–100% of the time, with the majority of studies reporting no pain treatment [12]. These findings are consistent with a prospective observational study over one week in 14 Canadian NICUs [7]. Predictors of the use of pharmacologic interventions (e.g., opiates) during tissue breaking procedures included being less ill at birth and receiving high frequency ventilator support, whereas parental presence significantly predicted the use of sweet taste or non-pharmacologic interventions (i.e., non-nutritive sucking, swaddling, rocking, positioning, skin-to-skin contact (SSC), breastfeeding) [7].

Poorly treated and/or prolonged pain exposure in preterm neonates has been linked to lasting consequences during a critical time in brain development. For infants delivered very preterm, controlling for gestational age, severity of illness and morbidity exposure to early pain-related distress is associated with both immediate physiologic instability and pain sensitivity as well as long-lasting deleterious impacts on cognition and behavior [13,14] and poor executive function and visual abilities [15]. A blunting of behavioural responses, increases in physiological responses, changes to pain thresholds, and alterations in hypothalamic-pituitary-adrenal axis development have been reported in relation to untreated pain exposure [14,16,17,18,19,20]. Moreover, reduction of brain growth in relation to pain exposure in infants 24–32 weeks gestational age (GA) during NICU admission, while controlling for other important factors such as GA at birth and severity of illness, has been shown [21]. Further studies have demonstrated that cumulative neonatal pain-related stress in very preterm neonates was associated with alteration in thalamic development [22], decreased frontal and parietal brain width, altered diffusion measures and functional connectivity in the temporal lobes, abnormalities in motor behaviour [23], and reduction in cerebellar size [24]. Emerging evidence suggests that procedural pain exposure in early life is also associated with epigenetic changes in infants born preterm [25,26].

This paper provides a narrative review of the current best evidence regarding the assessment and management of pain for infants born preterm requiring neonatal intensive care.

## 2. Assessing Pain in Preterm Infants

Most national and international guidelines on neonatal pain management declare pain assessment to be essential to achieve optimal pain and stress management [27,28,29,30]. The rationale for this is to identify situations when infants experience pain that should be treated and to avoid analgesic under- or overtreatment. A study from 243 European neonatal units showed that the link between assessment of continuous pain and the use of analgesic drugs was weak [31], suggesting that there was no assurance that preterm infants received effective pain alleviation.

The subjective and complex nature of pain makes pain assessment challenging. Self-evaluation of pain is the first choice for persons who can express themselves, verbally or by indicating the intensity of pain at some linear or pictorial scale, but for preterm infants, this is not an option. Instead, care providers must learn to recognize indicators of pain in infants and determine when they need pain alleviation based on these signs.

Signs of neonatal pain are categorized into behavioural, physiological, hormonal, or neurophysiological domains, which can either be observed individually or, preferably, in combination for better accuracy [32,33] (See Table 1). Since the pain experience is multi-dimensional, depending on both the intensity of the painful stimuli and on how it is processed and interpreted, a multi-dimensional approach to pain assessment is recommended [32]. In addition, other sources of non-pain related stress or discomfort can cause similar reactions, and those reactions can be dampened by analgesic or sedative drugs, or by illness. Thus, a combination of pain-signs should increase the specificity of pain assessment [34]. In the following we will outline several stand-alone pain-indicators and their combination in pain assessment tools. It should be noted that in many cases, how these indicators are expressed depends on the gestational age, illness, and alert state of the infant. For example, infants that are full-term, healthy, and awake will react with an up-regulation of the fight-and-flight system, whereas younger, sicker infants or infants in a deep sleep might instead react with dampened signs.

### 2.1. Observational Pain Assessment Scales

There are over 40 neonatal pain assessment scales published and even more constructed and used in clinical settings [32,43,44,45]. These are often divided into unidimensional scales that in their turn can be constructed from either single or multiple domains within the same dimension, or multidimensional scales combining items (see Table 2).

### 2.2. What Are the Most Sensitive and Specific Pain Assessment Tools?

A good pain assessment tool should detect all painful situations (high sensitivity), discriminate pain from non-painful situations (high specificity), measure a specific type of pain they are supposed to measure (good validity), give the same results when used repetitively or by different observers (good reliability), and, establish if a pain-relieving intervention was effective (good responsiveness) [63]. The choice of what pain assessment tool to use depends on the purpose, age of the population, and type of pain being assessed. A review of 352 clinical trials on neonatal pain revealed that 16% of the studies reported using pain scales that were not validated for the studied specific neonatal population or pain type [43], raising concerns regarding the validity of the findings. The clinical utility of the tool (ease of use in clinical settings) is also an essential component of consistent neonatal pain assessment. The most commonly used tool in clinical studies was PIPP/PIPP-R (154 studies), followed by NIPS (84 studies), NFCS (33 studies) and DAN (20 studies) [43].

### 2.3. Automatic Pain Assessment

There have been increasing attempts to use artificial intelligence and machine learning to analyze signals from preterm infants to evaluate if they indicate pain. An example is the ABC analyzer, which is an automated computer crying frequency analyzer that has shown correlation with the DAN-score during heel prick [64]. Despite numerous reports [65,66,67] to date, there is no system ready for clinical use.

### 2.4. Using Pain Assessment to Deliver Adequate Pain Alleviation

Several studies have examined the effects of implementing neonatal pain assessment, however, even if improved process outcomes have been seen, more research on the outcomes on patient level (i.e., if neonatal pain is alleviated) is needed [68]. In the clinical context there is a need for clear guidelines on how and when to assess pain, and above all, what to do with the results. Healthcare providers are recommended to follow an algorithm, or pathway, with different actions for different pain scores (e.g., to assess more often, use comfort measures, or give analgetic drugs) [69,70].

## 3. Management of Neonatal Pain

### 3.1. Breastfeeding and Breast Milk Feeding

Breastfeeding (direct latching of the neonate at the breast with active sucking and swallowing, ideally for at least two minutes prior to a painful procedure) and expressed breastmilk feeding have been studied for their pain reducing benefit. Direct breastfeeding may not be a feasible intervention in very preterm infants but should be considered in less preterm infants able to latch and suck at the breast. In the most recent Cochrane systematic review and meta-analysis, Shah et al. (2012) reported on findings from 20 studies examining the pain-reducing benefit of direct breastfeeding (10 studies *n* = 1075 infants) and expressed breastmilk feeding (10 studies, *n* = 996) during routine minor acute painful procedures in full-term infants [71]. Additionally, a subsequent systematic review reported on 15 studies (*n* = 1908) examining breastfeeding and expressed breastmilk feeding in full-term and preterm infants [72].

Direct breastfeeding has consistently been found to be more effective than comparator interventions, such as swaddling [71,73], maternal holding or SSC [74,75,76,77,78], topical anesthetics and cooling sprays [79,80], non-nutritive sucking [81], heel warming [82], music therapy [83], and placebo or no treatment [71,84,85]. Results are mixed when compared to sweet tasting solutions, however, direct breastfeeding has been found to be as or more effective [37,86,87,88,89,90]. Studies on the feeding of expressed breastmilk alone and without maternal contact report mixed results [72]. In those studies reporting a pain-reducing benefit of expressed breastmilk, sucking maturity [91] and combination of breast milk with other known pain-reducing strategies [92,93] were identified as factors that may contribute to the pain-reducing benefit.

The underlying pain-reducing mechanisms of direct breastfeeding are not fully elucidated. However, given the inconsistent effect of breastmilk provided independent of maternal contact, it is hypothesized that synergistic benefits of maternal closeness and SSC [94], maternal odor [95], auditory recognition [96], and sucking [97], contribute to the effectiveness of breastfeeding as a multi-modality pain-reducing intervention.

### 3.2. Skin-to-Skin Contact

Skin-to-skin contact (SSC) between an infant and mother (or alternative provider), first implemented as an alternative to incubator care to support temperature regulation and neonatal survival after preterm birth [98], was found to help increase time in quiet sleep state [99] and decreased crying time [100]. Given that quiet sleep state is associated with decreased pain response in early pain assessment studies [52,55], researchers began testing the efficacy of SSC for management of pain during acute procedures in preterm neonates.

In the most recent Cochrane systematic review on SSC for procedural pain in neonates, all studies (25 studies, *n* = 2001) reported favourable findings for SSC [94]. Of the 17 studies (*n* = 801) that compared SSC to standard care, significant reductions in heart rate during the painful procedure, crying time, and observational pain scores were found in a meta-analysis [94]. There is evidence that SSC is as or more effective than sweet tasting solutions [101,102,103], with SSC providing the same pain-reducing efficacy as sucrose and with sustained effect over repeated heel lance procedures for preterm infants in the NICU [104,105]. Additionally, one study examined neurodevelopmental impacts of providing maternal SSC compared to sucrose for repeated procedures across NICU hospitalization for preterm infants and demonstrated no difference between interventions [104]. Studies comparing maternal SSC with alternative provider (father, alternate female) SSC, have demonstrated that mothers are marginally more effective than fathers [106] and not significantly different from alternate female providers [107]. Based on current evidence [94], SSC should be supported as an efficacious way to reduce preterm infants’ procedural pain.

### 3.3. Adjuvant Physical Interventions

#### 3.3.1. Facilitated Tucking and Containment Interventions

Facilitated tucking is a physical containment intervention that involves placing hands on the head and limbs of an infant undergoing a painful procedure to maintain them in a side-lying flexed fetal position [108]. The most recent Cochrane systematic review and meta-analysis examining the pain-reducing effect of facilitated tucking [97] demonstrated that term and preterm infants who received facilitated tucking had reduced pain response immediately following painful procedures (15 studies, *n* = 445). There appears to be additional benefit of combining interventions.

Studies have shown that combinations of facilitated tucking and non-nutritive sucking with sucrose [109,110], facilitated tucking with sucrose [111], and facilitated tucking with non-nutritive sucking [112] were more beneficial compared to any of the interventions alone, in terms of reducing pain behaviours or recovery time after painful procedure. In this way, there appears to be additional benefit of combining interventions.

#### 3.3.2. Non-Nutritive Sucking

Oral stimulation through sucking on a pacifier has demonstrated pain-reducing properties in newborns, which is hypothesized to be a result of stimulation of oro-tactile and mechanoreceptors [113,114]. A Cochrane systematic review and meta-analysis of non-pharmacologic interventions for newborn procedural pain demonstrated that non-nutritive sucking improved regulation following painful procedures in both full-term and preterm neonates, and also improved pain reactivity in full-term neonates [97]. Combination of non-nutritive sucking with containment interventions [93] and oral sucrose [115] may improve pain reactivity and regulation following acute needle procedures.

#### 3.3.3. Combined Interventions

One question that has been raised includes whether combining non pharmacologic interventions could be beneficial. In a recent review, authors reported the findings of 14 studies, on the effectiveness of using sensorial saturation, defined as the combination of the use of oral sucrose with offered non-nutritive sucking, massage and caregiver voice, to reduce pain associated with medical procedures in full and preterm infants. Results indicated that while use of sucrose is likely the most salient component, use of a combined approach is likely associated with added benefit. The underlying modality of effectiveness is keeping with evidence supporting modalities encompassing multiple stimuli that involve gustatory, tactile, auditory, and olfactory mechanisms are generally more effective than single modalities.

### 3.4. Sweet-Tasting Solutions

Orally administered sucrose is the most extensively studied treatment for the management of neonatal procedural pain, with 74 randomized controlled trials (*n* = 7049) being included in the most recent Cochrane systematic review and meta-analysis examining its efficacy [115]. While various concentrations of sucrose have been tested (ranging from 10–50% concentration), the strongest evidence for the pain-reducing effect of 24% sucrose solution is when combined with non-nutritive sucking. In the three studies [116,117,118] combined in a meta-analysis (*n* = 278), significantly lower Premature Infant Pain Profile (PIPP) scores were observed for neonates who received 24% sucrose combined with non-nutritive sucking compared to water and non-nutritive sucking at 30-s following heel lance (WMD = −1.70, 95% CI [−2.13, −1.26]) and 60-s following heel lance (WMD = −2.14, 95% CI [−3.34, −0.94]) [115]. Combining sucrose with adjuvant interventions, such as non-nutritive sucking and swaddling, appears to provide added pain-reducing benefit. In their multi-center randomized controlled trial, Leng and colleagues (2015) enrolled 615 newborns to undergo heel lance in one of four intervention conditions: (1) 24% sucrose, (2) 24% sucrose plus non-nutritive sucking, (3) oral sucrose combined with swaddling, or (4) oral sucrose combined with both non- nutritive sucking and swaddling. A synergistic effect of sucrose and adjuvant interventions was found, with the combination of sucrose, non-nutritive sucking, and swaddling producing the most analgesic effects as measured by the revised Neonatal Facial Coding Scale, heart rate, and oxygen saturation [119].

Very few studies have examined the long-term effects of repeated sucrose administration. A systematic review of eight studies examining the efficacy and safety of repeated oral sucrose for procedural pain suggests that evidence is supportive of the safety and efficacy of repeated sucrose administration, however, results were mixed [120]. Pre-clinical studies using mouse models to mimic preterm neonatal pain and sucrose exposure in critical care suggests that exposure to repeated doses of 24% oral sucrose is associated with smaller brain volumes [121] and poorer short-term memory in adulthood compared to mice who received water [122]. One clinical study including 107 preterm infants born less than 31 weeks reported that infants who received greater than 10 doses of sucrose per day were prone to poorer attention and motor development outcomes [123]. Data on appropriate dosing of sucrose is limited, however, there is evidence to suggest that smaller volumes (0.1 mL, 0.2 mL) provide the same benefit in reducing pain using composite pain measures when compared to larger (e.g., 0.5–1.0 mL) sucrose volumes [124,125]. It is important to note that sucrose should be administered to the tip of the tongue 2 min prior to the procedure, may be repeated throughout the procedure and should not be given via an oral or naso-gastric tube.

### 3.5. Pharmacological Interventions for Neonatal Pain

Despite significant progress that has been made over the past three decades addressing the prevention, assessment, and management of neonatal pain, many questions remain regarding optimal use of pharmacological agents. Lack of efficacy data and uncertainty regarding long-term outcomes are two of the primary research gaps that clinicians face when attempting to manage neonatal pain [126]. Moreover, the optimal approach to the management of pain-related distress also remains unclear. Despite these uncertainties, recognition of the significant adverse effects of untreated pain in early life and some existing evidence has led to the consistent inclusion of pharmacologic agents in pain prevention and management guidelines for neonates [28,127,128,129].

The most used non-opioid analgesic to manage pain in children including neonates is paracetamol (acetaminophen). While commonly used in older children and adult populations, non-opioid agents such as NSIADs, gabapentin, and dexmedetomidine are contraindicated or less commonly used in neonatal populations. Local topical and regional agents are also utilized with variable uptake across neonatal units, while the mainstay for post-operative procedures includes opioids such as morphine and fentanyl. Adjuvant agents are also used for the management of pain-related distress and refractory pain. An overview of the use of these agents is included below.

#### 3.5.1. Non-Opioid Analgesics

##### Paracetamol

Based on current best evidence, paracetamol does not provide effective pain relief for infants undergoing procedural pain, despite its frequent use [130,131]. In a Cochrane review of eight studies including 614 infants, paracetamol when compared with water, cherry elixir, or Eutectic Mixture of Local Anesthetics (EMLA) cream did not reduce pain associated with heel lance nor reduce pain associated with examinations [130]. There is some evidence that paracetamol has opioid-sparing effects for major pain and post-operative conditions and is effective in treating minor to moderate pain conditions [126]. A recent consensus statement from the Enhanced Recovery After Surgery (ERAS) Society recommends that unless contraindicated, regular dosing (not on an ‘‘as needed’’ basis) of paracetamol following neonatal intestinal surgery during the early postoperative period (to minimize but not replace opioid use) should be used [132].

###### Alternative Non-Opioid Agents

Despite the widespread use of non-steroidal anti-inflammatory drugs (NSAIDs) to treat pain in older pediatric populations, their use for neonatal populations is less common. Given the risk associated with renal injury and gastrointestinal bleeding, most NSAIDs such as ketorolac are not approved for use in infants less than six months of age, with greatest risk reported in infants less than three weeks of age and those delivered preterm [133].

Given the positive benefits of pain relief and opioid sparing in older children and adults, there is increasing interest in the use of dexmedetomidine and gabapentin to treat postoperative pain and pain-related distress in neonates. Their use remains off-label, given the lack of FDA approval, in neonatal populations. To date, only a few small studies have been reported. Dexmedetomidine has been associated with less respiratory depression or gastrointestinal dysmotility when compared to fentanyl [134], and there is some suggestion that the use of alpha-2 agonists such as dexmedetomidine may be neuroprotective [135]. Gabapentin, a g-aminobutyric acid analogue, has been reported to be potentially beneficial in treating irritability and refractory pain-related distress in neonates [136]. Despite these promising findings, further study regarding their efficacy and safety are warranted before routine use can be recommended.

#### 3.5.2. Local Anesthetics

##### Regional

There is increasing interest in the use of spinal and epidural anesthesia for postoperative pain management in neonates. Local agents such as bupivacaine, lidocaine, or ropivacaine can be administered as regional anesthesia in awake patients for minor surgeries such as inguinal hernia repair and circumcision, or for procedures such as chest tube placement [137,138]. The use of regional anesthesia has been associated with reduced apnea and bradycardia in the postoperative period but was not associated with a decrease in postoperative opioid use [138]. Variability in availability of skilled practitioners has been cited as one of the reasons that this approach is underused [139].

###### Topicals

Considerable variation in degree of efficacy regarding the use of topical anaesthetics in neonates has been reported across procedures [140]. There is some evidence that the use of topical anesthetic eye drops reduces pain associated with routine eye examinations and screening conducted in the NICU [141], although its use does not provide optimal pain relief, and as such should be used in combination with sweet tasting solution or an effective non-pharmacologic intervention [142]. Additionally, topical agents such as EMLA, a combination of 2.5% prilocaine and 2.5% lidocaine, has been shown to reduce pain associated with lumbar puncture at the time of needle insertion [143], although caution regarding the application is required especially in very preterm infants [140]. While topical anaesthetics have demonstrated some effectiveness for reducing circumcision-related pain [144,145,146,147], a recent review of 29 randomized trials demonstrated that topical anesthetics used as a single agent provided insufficient pain relief [148]. There is also strong evidence that topical anesthetics are ineffective in reducing the pain associated with heel lance [149,150], venipuncture [151], insertion of intravenous or intraarterial lines [152,153], and frenotomy [154].

#### 3.5.3. Opioids

Opioids are the mainstay for the effective treatment of moderate to severe pain across all ages, including neonates. The most commonly used agents for neonatal populations include morphine and fentanyl. Despite the benefits, safe and effective dosing of systemic drugs (specifically opioids) for pain relief in neonates is challenging, as their neurodevelopmental stage makes them highly sensitive to drug effects [155] and can demonstrate slow drug clearance [156,157,158,159]. Opioid overuse, especially in very preterm infants requiring prolonged ventilation, has been associated with adverse effects. Higher exposure to morphine has been associated with higher incidence of death, intraventricular hemorrhage or periventricular leukomalacia, hypotension, prolonged ventilation, and feeding intolerance [160,161,162,163], as well as reduced cerebellar volume and poorer 18-month motor and cognitive scores [24], when compared with lower morphine exposure.

It is somewhat reassuring to note that studies examining the longer-term impact of morphine exposure in ex-preterm infants at age five [164], eight, and nine [165] did not report adverse neurological effects. Additionally, higher dosages of continuous infusion of fentanyl has been associated with decreased cerebellar growth [166] and poorer neurodevelopmental outcome at two years of age [167] in contrast to the use of lower dosing [156].

One significant question which remains unanswered relates to the optimal administration of opioids or adjuvant therapies, either as a continuous infusion or intermittent bolus, to achieve effective pain reduction and reduce cumulative opioid exposure and risk for adverse outcome. In the few studies addressing this question, results are mixed. In post-operative infants (*n* = 83), while there were no group differences in oxygen saturations levels, infants receiving intermittent boluses demonstrated greater pain scores indicating distress (32 vs. 13%, *p* < 0.001) compared to infants receiving continuous morphine infusions of 20 ng/mL. In a randomized trial comparing the pharmacokinetics of fentanyl across 100 neonates, serum fentanyl concentrations were steady in the infants receiving a continuous infusion (1 mcg/kg loading dose followed by 1 mcg/kg/hour infusion) in contrast to wide fluctuations in serum concentration with high-peak concentrations in infants receiving bolus administration (1 mcg/kg/dose administered every four hours) [168]. Fentanyl bolus administration (1–2 μg/kg/dose) prior to procedural pain was not found to significantly affect cerebral oxygenation, cerebral tissue oxygen extraction, or cardiac output in stable preterm infants (*n* = 28) [169]. Similarly, in a large national cohort study examining the association of early continuous infusions of opioids and/or midazolam with survival and sensorimotor outcomes at age two years in very premature infants who were ventilated, infants either received continuous opioid and/or midazolam infusion in the first week of life (*n* = 450) or no treatment (*n* = 472). Infants in the treated group had improved survival without any difference in moderate or severe sensorimotor impairments at age two years [170]. More research is needed to determine the best practices for safe and effective administration of opioids for pain management in neonates.

### 3.6. Families and Pain Management

Modern neonatal care is family-centered and supports parental presence in the neonatal unit and parental involvement in care procedures, including pain management, with proper counselling and information [171,172,173,174] Historically, the parent’s role in pain care has been significantly underutilized, and there has been a lack of focus on ensuring families have the resources they need to best help manage their infant’s pain. Studies show that parents can recognize their infant’s pain but tend to underestimate it compared to healthcare professionals [175,176]. A parent who can be with their newborn infant continuously will be able to learn its signs of comfort and discomfort and should be involved in pain assessment in collaboration with the staff [177].

There is increasing recognition that parents are aware of their infant’s pain. Although this awareness is stressful for parents [178,179], they want to be informed and contribute to preventing and alleviating that pain [180]. The important role parents can play in reducing the pain their preterm infant endures also strengthens their parental role [181]. Many non-pharmacological pain-alleviating methods are best or even exclusively performed by parents, such as SSC, facilitated tucking, or breastfeeding [182]. Neonatal healthcare professionals should guide parents, through provision of knowledge and resources, to empower families to be both advocates and active participants for their preterm infant’s pain care [177,183], and fully engaged members of the interprofessional healthcare team [184,185].

## 4. Conclusions

Despite an increased awareness that newborn infants experience pain, which can lead to short- and long-term negative health outcomes, management of this pain remains varied with mixed results on pain alleviation. Validated and appropriate pain assessment tools should be implemented as a standardized approach for healthcare providers treating neonates to guide pain-relieving treatments, including pharmacological and non-pharmacological interventions. Finally, it is important to engage families in the pain management of newborns, as parents are a valuable resource who want to be involved in alleviating the pain of their infant.

## Figures and Tables

**Table 1:** Indicators of neonatal pain.

Category	Description
Behavioural signs	
*Alertness and sleep-wake state*	A hyper-alert state is often seen during on-going or prolonged pain Younger or sicker infants can need longer time to comfort themselves and return to rest after a painful event
*Body movements and muscle tension*	Full-term infants withdraw limbs from pain, whereas in preterm infants these responses are more diffuse and harder to control Strong infants can react with increased muscle tension in arms and legs Younger or sicker infants can turn flaccid
*Crying*	Healthy, awake, and full-term newborn infants react with moaning or crying that gets more intense and with longer duration with increasing discomfort. In contrast, infants of lower gestational age, sick, in a sleeping state, or sedated will have difficulties showing a vocal reaction so the absence of crying must not be taken for absence of pain; crying can often be noted as a weaker moaning sound
*Facial activity, grimacing*	Most used facial expressions that are indicators of pain: ○ Brow bulge ○ Squeezed eyes ○ Distinct furrows from the nose to the ends of the mouth ○ Tense and stretched mouth and tongue ○ Raised cheeks
Physiological signs	
*Heart rate and heart rate variability (HRV)*	In healthy full-term infants, pain triggers an increased heart rate which leads to increased blood pressure and a red skin color Conversely, younger and sicker infants may react with an increase or decrease in heart rate, possibly bradycardia Pain reduces HRV HRV is best suited for assessing prolonged pain
*Respiration rate*	In robust infants respiration rate will increase as a result of acute pain, whereas the opposite, and even apneas can be seen in preterm infants who do not have energy to trigger the fight-or-flight system Pain in the thorax-region can lead to impaired breathing
*Oxygen saturation*	Changes in oxygen saturation follows pain-associated changes in respiration and heart rate Both an increase and decrease of oxygen saturation can be seen, depending on the context
*Cortisol*	Increased levels can be seen after surgery [35] and painful procedures [36] Measured in plasma, saliva, or urine High variability, more useful in research than clinically
Neurophysiological signs	
*Cerebral oxygenation*	Pain measured with Near Infrared Spectroscopy (NIRS) associated with cerebral activation and changes in oxygenated and deoxygenated haemoglobin concentration in the brain An increase in oxygenated haemoglobin is assumed to reflect neuronal activation caused by pain NIRS is sensitive to movement artefacts [37,38]
*EEG, evoked potentials*	Multi-channel EEG used as a proxy for neuronal activity with the majority of studies reporting on pain-related event-related potentials during acute painful procedures [39,40] Primarily used in research, no standard clinical pain assessment method/tool
*Functional magnetic resonance imaging (fMRI)*	Has shown which areas in the infant brain are activated by pain [41,42] Not relevant as a bedside pain assessment tool Can help in developing and validating other measures

**Table 2:** Pain Assessment Tools for Term and Preterm Infants.

Acronym/Name	Items
Acute Procedural Pain	
***ABC*** [46]	Acuteness, rhythmicity, and continuity of crying
***BIIP*** [47] *Behavioral Indicators of Infant Pain*	Behavioral state, brow bulge, eye squeeze, naso-labial furrow, horizontal mouth stretch, taut tongue, finger splay, fisting
***BPSN*** [48,49] *Bernese Pain Scale for Neonates*	Alertness, duration of crying, time to calm, skin colour, eyebrow bulge with eye squeeze, posture, breathing pattern
***DANS*** [43], *Douleur Aigue Nouveau-ne*	Facial expression, limb movements, vocal expression
***FANS*** [50] *Faceless acute neonatal pain scale*	HRV, acute discomfort, limb movements, vocal expressions
***FLACC*** [51] *Face, Legs, Activity, Cry, Consolability scale* *(validated birth to adolescence)*	Face, legs, activity, cry, consolability
***NFCS*** [52] *Neonatal Facial Coding System* *(some validation for post-operative pain)*	Brow bulge, eye squeeze, naso-labial furrow, open lips, horizontal mouth stretch, vertical mouth stretch, lip purse, taut tongue, chin quiver
***NIPS*** [53] *Neonatal Infant Pain Scale*	Facial expression, cry, breathing patterns, arms, legs, state of arousal
***NIAPAS*** [54] *Neonatal Infant Acute Pain Assessment Scale*	GA, alertness, facial expressions, crying, muscle tension, breathing, respirator/CPAP, reaction to handling, heart rate, oxygen saturation
***PIPP, PIPP-R*** [55,56] *Premature Infant Pain Profile—Revised*	GA, behavioural state, heart rate, oxygen saturation, brow bulge, eye squeeze, naso-labial furrow
Post-operative Pain	
***N-PASS*** [57] *Neonatal Pain, Agitation and Sedation* *Scale (some validation for acute procedural pain)*	Crying/irritability, behavior state, facial expression, extremities tone, vital signs
***PAT*** [58] *Pain Assessment Tool*	Posture/tone, sleep pattern, expression, colour, cry, respirations, heart rate, oxygen saturation, blood pressure, nurse’s perception
Prolonged Pain	
***ALPS-Neo*** [59] *Astrid Lindgren and Lund Children’s Hospital’s Pain and Stress Assessment Scale for Preterm and Sick Newborn Infants*	Facial expression, breathing pattern, tone of extremities, hand/foot activity, level of activity
***COMFORT neo*** [60] *(Also validated for sedated infants)*	Alertness, calmness/agitation, respiratory response, crying, body movement, facial tension, body muscle tone
***EDIN*** [61] *Échelle Douleur Inconfort Nouveau-né*	Facial activity, body movements, quality of sleep, quality of contact with nurses, consolability
***MAPS*** [62] *Multidimensional Assessment of Pain Scale* *(Validated Birth to 31 months)*	Vital signs, breathing pattern, facial expressions, body movement, state of arousal
